# Unexpected Recovery: A Report on the Spontaneous Regression of a Herniated Cervical Disc

**DOI:** 10.7759/cureus.41429

**Published:** 2023-07-05

**Authors:** Sara Aljohani, Maryam Alshanqiti, Moajeb Alzahrani

**Affiliations:** 1 Collage of Medicine, King Saud Bin Abdulaziz University for Health Sciences, Jeddah, SAU; 2 Department of Neurosurgery, King Fahad General Hospital, Madinah, SAU; 3 Department of Neurosurgery, King Abdulaziz Medical City, Ministry of National Guard Health Affairs, Jeddah, SAU; 4 College of Medicine, King Saud Bin Abdulaziz University for Health Sciences, Jeddah, SAU

**Keywords:** spontaneous regression, cervical disc herniation, magnetic resonance imaging, herniated disc, cervical disc

## Abstract

We describe a case in which a herniated cervical disc was compressing the spinal cord. Surgical treatment was offered based on the patient's symptoms and magnetic resonance imaging (MRI), but the patient declined. The patient's symptoms were relieved after 10 months of nonsurgical intervention, and a subsequent MRI revealed that the cervical disc herniation (CDH) had regressed. This phenomenon is well established in the lumbar region but remains rare in the cervical spine. We recommend opting for conservative management and frequent follow-ups for patients with CDH unless they present with a surgical urgency.

## Introduction

Cervical disc herniation (CDH) is a condition characterized by displacement of the nucleus pulposus of the intervertebral disc in the cervical region, causing spinal cord compression or nerve root impingement. The symptoms can vary depending on the magnitude of the herniation and cervical levels affected [1]. The manifestation of symptoms related to CDH is predominantly observed in individuals who are above the age of 40. Furthermore, individuals engaged in certain professions or hobbies that require heavy lifting and axial loading are at a higher risk of developing this condition [2].

The management of such cases can either be conservative or surgical with the latter being reserved for cases that fail conservative interventions and/or present with signs or symptoms of motor deficit [3]. There are few reported cases of CDH resolving spontaneously without any intervention [4,5]. This phenomenon is well established in the lumbar region, yet the data on cases occurring in the cervical region remain limited. Hence, we present a rare case of a significant regression of a large CDH, presenting with neck pain and left radiculopathy, without any intervention.

## Case presentation

**Figure 1:**
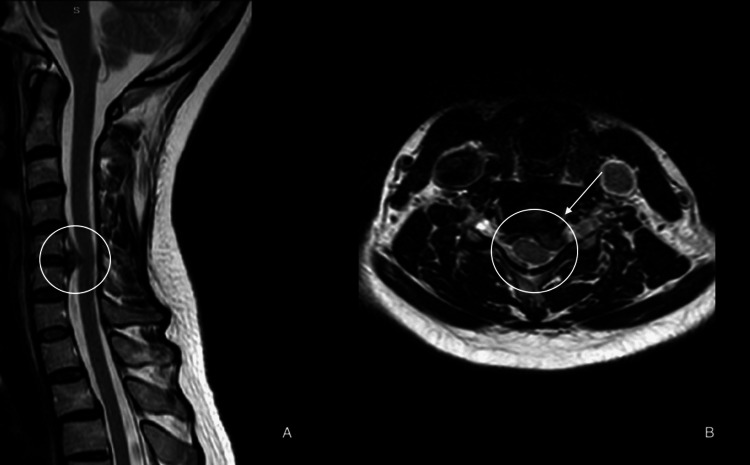
(A) Sagittal and (B) axial T2-weighted MRI of the cervical spine showing a large C5-C6 foraminal/paracentral disc protrusion with canal stenosis. MRI, magnetic resonance imaging

A 47-year-old female presented to the clinic with severe progressive neck pain and left upper limb radiculopathy that started three weeks ago. The pain mildly improved with analgesia. She denied weakness, sensory deficits, urinary incontinence, or a history of trauma. Upon examination, the patient displayed neurological intactness, with 5/5 motor power in all limbs, intact sensation, normal reflexes, and a negative Hofmann sign. A cervical MRI, which was done in another institution upon the onset of symptoms, showed a large left paracentral/foraminal C5-C6 disc prolapse and severe canal/foraminal stenosis (Figure 1).

**Figure 2:**
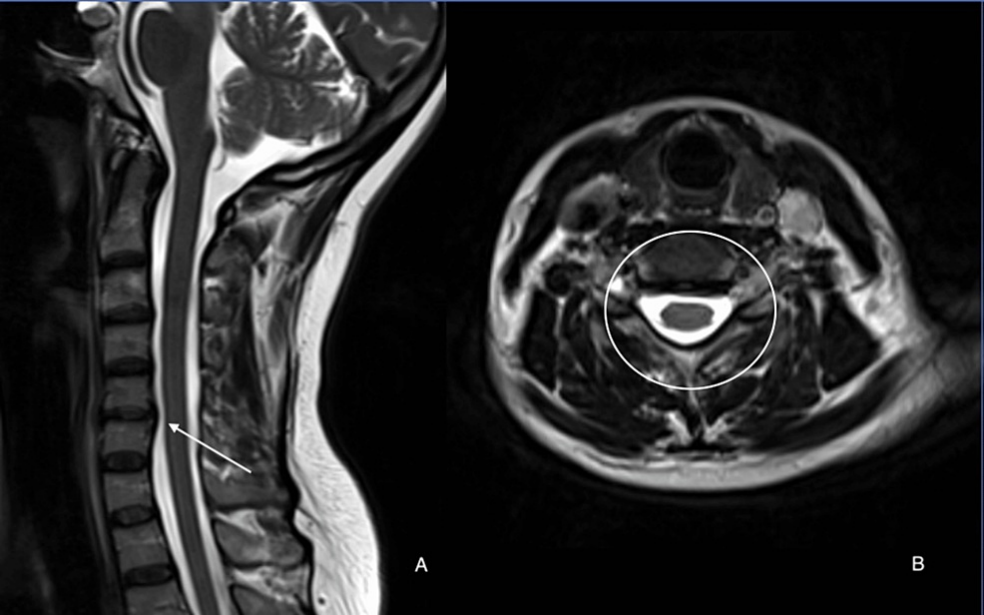
Repeat cervical MRI done 10 months after presentation showing regression of the previously documented cervical disc. MRI, magnetic resonance imaging

The patient was offered surgery, and after hearing the benefits and risks, she opted for conservative treatment instead, including nonsteroidal anti-inflammatory drugs (NSAIDs), gabapentin, and muscle relaxant. On regular follow-ups, the patient’s symptoms remained stable, with no increase in her pain and no new neurological deficits. She mentioned that the pain was manageable via analgesia and was advised to seek physiotherapy. A repeat MRI was done 10 months after the initial presentation as part of the follow-up plan. The results indicated a resolution of the extruded fragment, with only minimal foraminal narrowing observed, as depicted in Figure 2. Then, four months after that the patient was completely asymptomatic.

## Discussion

CDH is a condition commonly caused by a combination of degenerative changes in the spine, trauma, or an underlying medical condition such as ankylosing spondylitis. Symptoms of CDH include neck pain, radiculopathy, and myelopathy. Treatment typically consists of conservative management such as physical therapy, medications, and possibly surgical intervention [1-3]. Spontaneous regression of CDH is the process by which the spinal disc herniation shrinks or disappears without any medical intervention.

The rare phenomenon of spontaneous regression of CDH has been the subject of multiple studies, with its etiology believed to be multifactorial. In a review conducted by Sharma et al. [4], the authors analyzed published articles involving patients with MRI-confirmed regressed CDHs. The study population demonstrated an equal distribution of males and females, with an average age of 41 years, signifying middle adulthood. Commonly reported symptoms among these patients were neck pain and radiculopathy, while a smaller proportion presented with myelopathy. The herniated discs were predominantly located in the paracentral or foraminal regions and exhibited a higher incidence of spontaneous regression than central lesions. The most frequently affected disc levels were C5-C6, with extruded or sequestrated herniations being the majority. The average time interval between the initial presentation and the documented spontaneous regression on successive MRI scans was one year or less. These findings imply that factors such as lesion location, disc level, and herniation type may significantly impact the likelihood of spontaneous cervical disc regression [4].

Furthermore, larger or extruded CDH may regress more rapidly than smaller or protruded ones, as they have a greater probability of penetrating the annulus fibrosus and posterior longitudinal ligament, exposing them to the systemic circulation within the epidural space [5]. The pathophysiology of disc regression remains uncertain, with three proposed mechanisms for the spontaneous regression of CDHs. One theory suggests that herniated discs retract back into the intervertebral space, while another posits that the herniated fragment gradually disappears due to dehydration and shrinkage [6-9]. A third hypothesis involves an inflammatory response, neovascularization, and phagocytosis triggered by a herniated disc in the epidural region, which leads to the immune system perceiving the extruded disc as a foreign body [6,10]. 

CDH typically presents with radiculopathy resulting from cervical root compression and/or myelopathy due to spinal cord compression. In neurosurgical practice, surgical intervention is often favored for patients with persistent radiculopathy or myelopathy [4]. Although cervical disc surgery is relatively safe and can lead to rapid and complete symptom improvement, the possibility of spontaneous regression implies that conservative therapy may be a successful treatment option for many cervical herniations [4,11]. The limited number of reports in the literature suggests that conservative treatment for herniated cervical discs may be a viable management option, particularly for patients with neuroforaminal herniations and no neurological deficits. However, closely monitoring these patients is crucial to detect neurological deterioration. Consequently, the risk of neurological worsening should be carefully assessed before opting for conservative management.

## Conclusions

This case report and previous studies on cervical disc regression indicate that non-surgical treatment can serve as a safe and effective alternative for patients with radiculopathy or even myelopathy, provided they do not have severe neurologic deficits or require immediate surgery. When considering conservative care for patients with CDHs, age, disc location, size, and type of herniation should be taken into account as potential predictors of regression.
